# Supplementary material for: Mechanosensitive Channel PIEZO1 Senses Shear Force to Induce KLF2/4 Expression via CaMKII/MEKK3/ERK5 Axis in Endothelial Cells
Source: Cells. 2022 Jul 13;11(14):2191. doi: 10.3390/cells11142191 (PMC9317998; doi:10.3390/cells11142191)
Supplement: Supplementary file 1 [file cells-11-02191-s001.zip › cells-1778112-supplementary.pdf]

Supplementary Materials

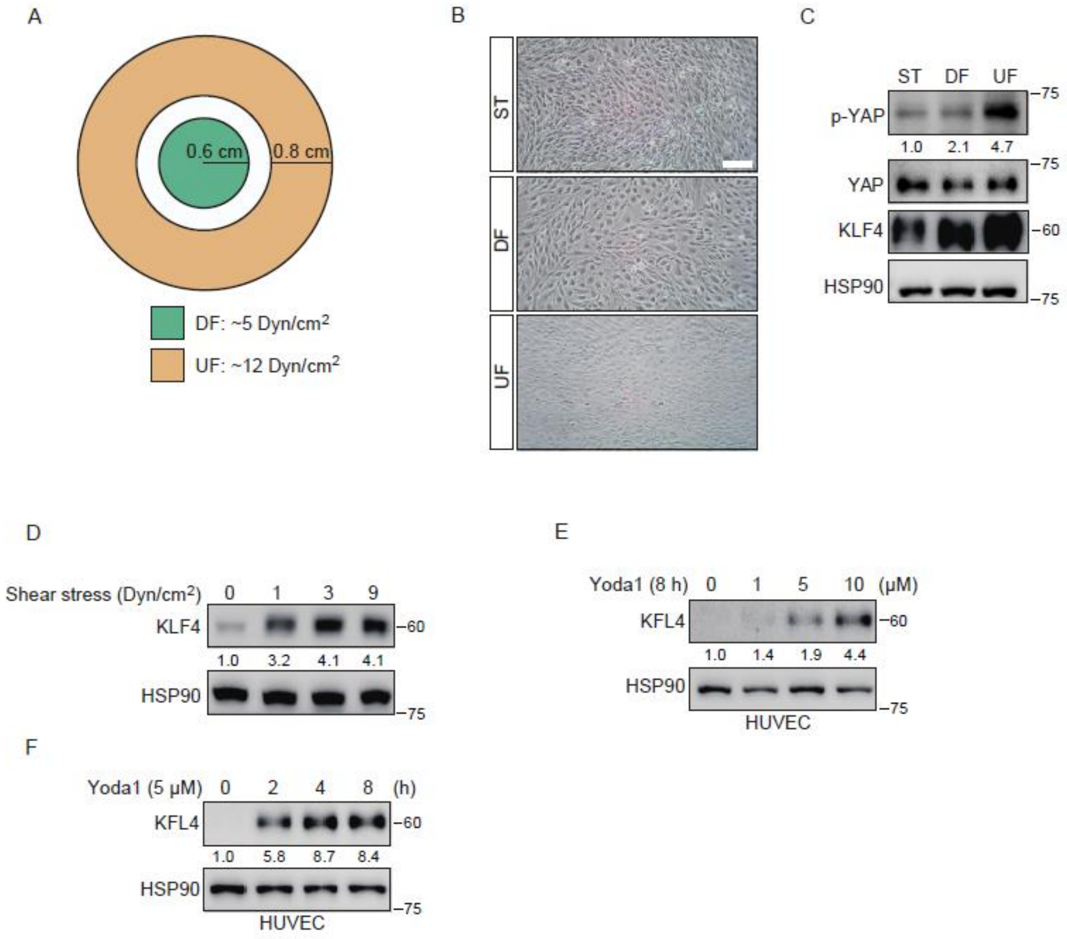

Figure S1. KLF2/4 expression is increased under laminar shear stress using a flow model in vitro. (A) Diagram of differential patterns of shear stress exposure. Cells were harvested throughout the entirety of the well without shear stress application (static, ST), or respectively in the center or periphery of the well after shear stress exposure. DF: disturbed flow. UF: unidirectional laminar flow. (B) MBMECs were exposed to differential shear stress for 5 d and morphology was analyzed under a microscope. Scale bars, 50 μm. (C) MBMECs were exposed to differential shear stress for 5 d and cell lysates were probed with the indicated antibodies. (D) MBMECs were exposed to UF with indicated rotating speed of orbital shaker and KLF4 proteins were monitored by western blotting. (E) HUVECs were treated with Yoda1 with the indicated concentration for 8 h and KLF4 proteins were monitored by western blotting. (F) HUVECs were treated with Yoda1 (5 μM) for the indicated time and KLF4 proteins were monitored by western blotting.

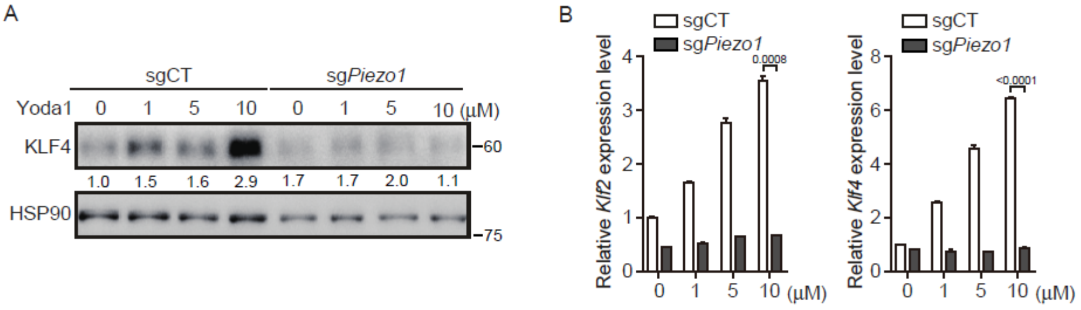

Figure S2. Piezo1 deficiency restrains Yoda1-induced KLF2/4 expression (A) Control and Piezo1-dificient MBMECs cells were treated with Yoda1 with the indicated concentration for 8 h and KLF4 proteins were monitored by western blotting. (B) Control and Piezo1-dificient MBMECs cells were treated with Yoda1 with the indicated concentration for 2 h and mRNA levels of Klf2 and Klf4 were measured by quantitative RT-PCR. Data are representative of three independent experiments and presented as mean ± SEM of three technical replicates.

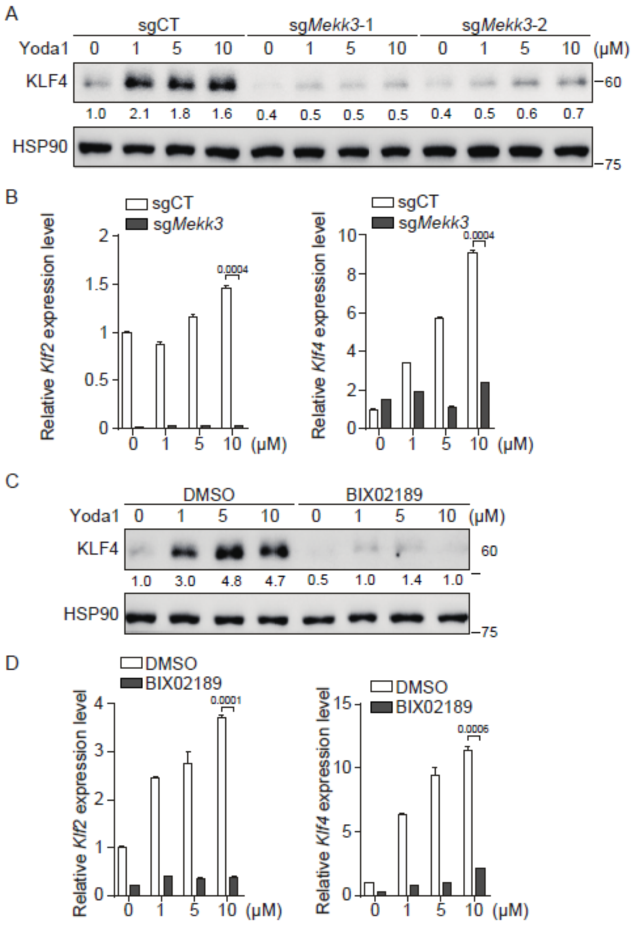

Figure S3. MEKK3/MEK5/ERK5 signaling pathway is involved in PIEZO1-induced KLF2/4 expression**.** (A) Control and *Mekk3*-dificient MBMECs cells were treated with Yoda1 with the indicated concentration for 8 h and KLF4 proteins were monitored by western blotting. (B) Control and *Mekk3*-dificient MBMECs cells were treated

with Yoda1 with the indicated concentration for 2 h and mRNA levels of *Klf2* and *Klf4* were measured by quantitative RT-PCR. (C) MBMECs were pretreated with MEK5 inhibitor (BIX02189, 10 μM) for 12 h, and then treated with Yoda1 with the indicated concentration for 8 h and KLF4 proteins were monitored by western blotting. (D) MBMECs were pretreated with MEK5 inhibitor (BIX02189, 10 μM) for 12 h, and then treated with Yoda1 with the indicated concentration for 2 h and mRNA levels of *Klf2* and *Klf4* were measured by quantitative RT-PCR. Data are representative of three independent experiments and presented as mean ± SEM of three technical replicates by an unpaired Student's t-test (B, D). ***p < 0.001.

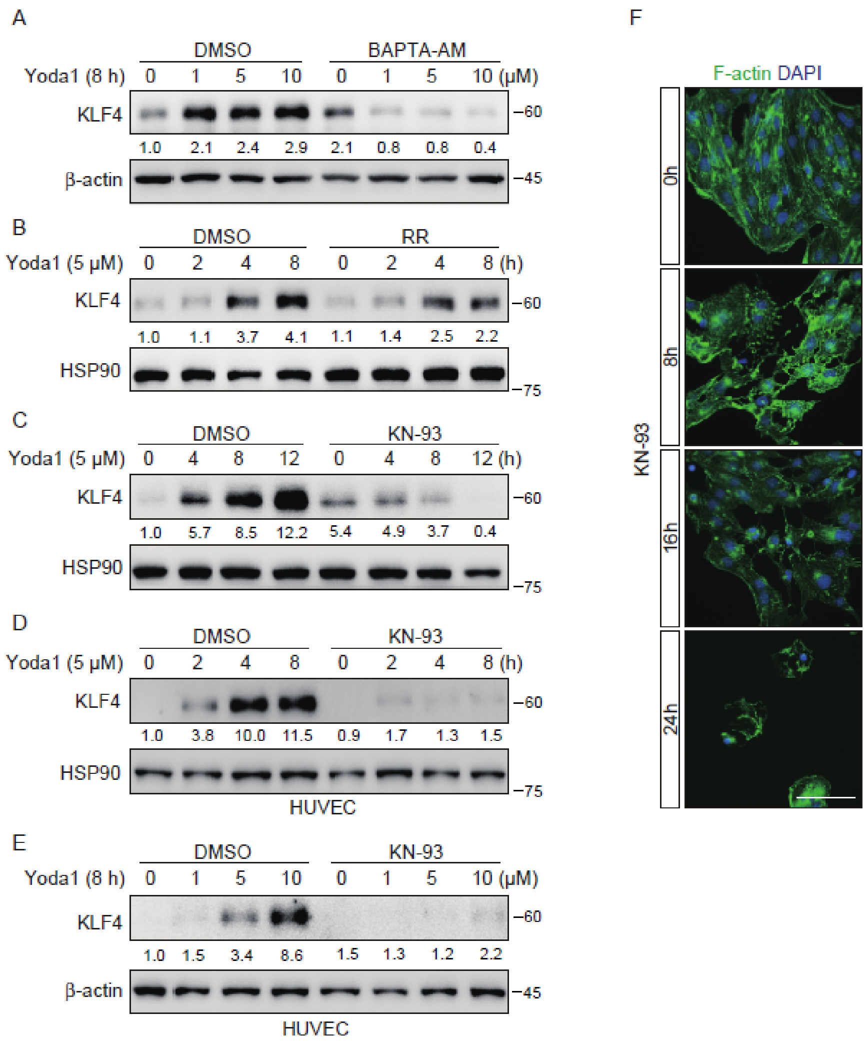

Figure S4. CaMKII is required for Yoda1-induced KLF4 expression,and maintains endothelial cytoskeleton. (A) MBMECs were pretreated with BAPTA-AM (10 μM) for 2 h, and then treated with Yoda1 with the indicated concentration for 8 h. KLF4 proteins were monitored by western blotting. (B) MBMECs were pretreated with RR (10 μM) for 2 h, and then treated with Yoda1 (5 μM) for the indicated time. KLF4 proteins were monitored by western blotting. (D) HUVECs were pretreated with KN-93 (10 μM) for 2 h, and then treated with Yoda1 (5 μM) for the indicated time. KLF4 proteins were monitored by western blotting. (E) HUVECs were pretreated with KN-93 (10 μM) for 2 h, and then treated with Yoda1 with the indicated concentration for 8 h. KLF4

proteins were monitored by western blotting. (F) Representative phalloidin staining for F-actin (green) and nuclei (blue) of MBMECs treated with KN-93 (10 μM) for the indicated time. Scale bars, 50 μm.

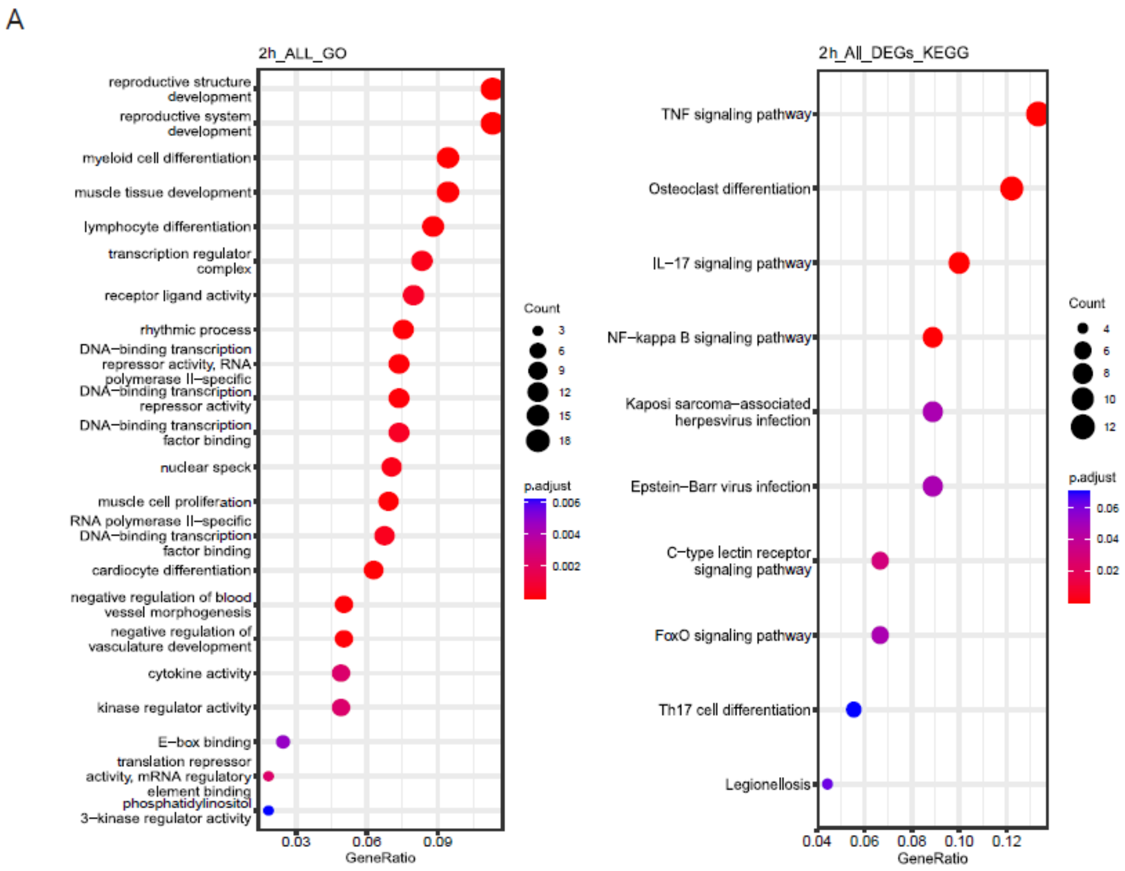

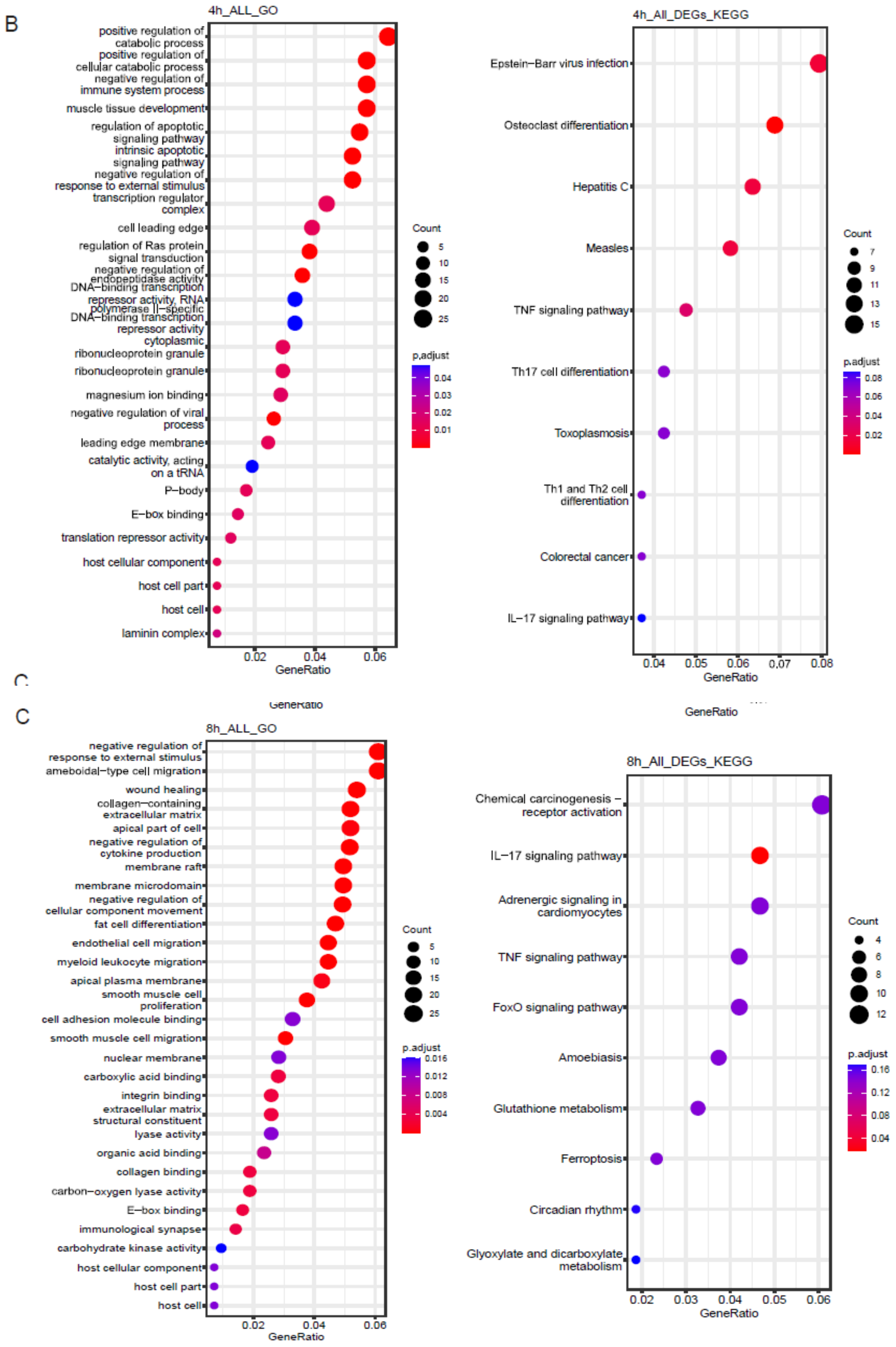

Figure S5. The analysis of enriched gene ontology and KEGG signaling pathway in MBMECs treated with Yoda1 in a time-course manner. (A-C) The enriched Gene ontology and KEGG signaling pathways were selected to demonstrate biological functions and related signaling pathways among DEGs. The horizontal axis indicates

gene ratio. Colors represent the significance of differential enrichment; the size of the circles represents the number of genes.

Supplementary Table S1. siRNA and guide RNA target sequence

siRNA	Target sequence
m*Klf2* siRNA#1	GCACGGATGAGGACCTAAA
m*Klf2* siRNA#2	ACAACGTGTTGGACTTCAT
m*Klf4* siRNA#1	ACAACGTGTTGGACTTCAT
m*Klf4* siRNA#2	CGGAGTTGGACCCAGTATA
sgRNA	Target sequence
m*Piezo1* gRNA#1	AGCATTGAAGCGTAACAGGG
m*Mekk3* gRNA#1	GCACAAGTAGACCCTGCCGA
m*Mekk3* gRNA#2	GCTCCCATCAATTGGCGTCG

Supplementary Table S2. Antibody information

Name	Source	#Catalog	Dilution
Rabbit Anti-KLF2	Merck	09-820	WB (1:500)
Rabbit Anti-KLF4	Abcam	ab214666	WB (1:1,000)<br>IF (1:100)
Rabbit Anti-KLF4	Abcam	ab215036	WB (1:1,000)
Rabbit Anti-CD31	Abcam	ab182981	IF (1:200)
Rabbit Anti-PIEZO1	Proteintech	15939-1-AP	WB (1:500)
Rabbit Anti-MEKK3	CST	5727	WB (1:1,000)
Rabbit Anti-Phospho-ERK5	CST	12950	WB (1:500)
Rabbit Anti-ERK5	CST	3371	WB (1:500)
Rabbit Anti-Phospho-CaMKII	CST	12716	WB (1:500)
Rabbit Anti-CaMKII	Abcam	ab52476	WB (1:500)
Mouse Anti-Flag	Sigma	F3165	WB (1:1,000)<br>IF (1:100)
Rabbit Anti-HA	Proteintech	51064-2-AP	WB (1:1,000)<br>IF (1:100)
Rabbit Anti-Phospho-p65	CST	3033	WB (1:1,000)
Mouse Anti-p65	CST	6956	WB (1:1,000)
Rabbit Anti-Phospho-YAP	CST	13008	WB (1:1,000)
Rabbit Anti-YAP	Proteintech	13584-1-AP	WB (1:1,000)
Rabbit Anti-HSP90	Proteintech	13171-1-AP	WB (1:2,000)
Rabbit Anti-β-actin	ABclonal	AC026	WB (1:50,000)

Supplementary Table S3. qPCR primers

Name	Sequence
m*Klf2*-F	TGCGTACACACACAGGTGAGA
m*Klf2*-R	CAGGCTACATGTGTCGCTTCA
m*Klf4*-F	TACCCCTCTCTCTTCTTCGGA
m*Klf4*-R	TTCCTCACGCCAACGGTTAGT

m*Piezo1*-F	GCCTGTCCACCGTGTGGACCTG
m*Piezo1*-R	CCGCACCCCAAACCAGTTGGC
m*A20*-F	AGCAAGTGCAGGAAAGCTGGCT
m*A20*-R	GCTTTCGCAGAGGCAGTAACAG
m*Gapdh*-F	CATCACTGCCACCCAGAAGACTG
m*Gapdh*-R	ATGCCAGTGAGCTTCCCGTTCAG
m*Rn18s*-F	GCCGCTAGAGGTGAAATTCTT
m*Rn18s*-R	CGTCTTCGAACCTCCGACT
